# Structure–function analysis of PorX_Fj_, the PorX homolog from *Flavobacterium johnsioniae*, suggests a role of the CheY-like domain in type IX secretion motor activity

**DOI:** 10.1038/s41598-024-57089-9

**Published:** 2024-03-19

**Authors:** Mariotte Zammit, Julia Bartoli, Christine Kellenberger, Pauline Melani, Alain Roussel, Eric Cascales, Philippe Leone

**Affiliations:** 1 Laboratoire d’Ingénierie des Systèmes Macromoléculaires (LISM, UMR7255), Institut de Microbiologie de la Méditerranée, Aix Marseille Univ, Centre National de la Recherche Scientifique, Marseille, France; 2 Laboratoire de Chimie Bactérienne (LCB, UMR7283), Institut de Microbiologie de la Méditerranée, Aix Marseille Univ, Centre National de la Recherche Scientifique, Marseille, France

**Keywords:** Type IX secretion system, *Flavobacterium johnsoniae*, Two-component system, Response regulator, Crystal structure, CheY, PglZ, Bacterial structural biology, Bacterial pathogenesis, X-ray crystallography

## Abstract

The type IX secretion system (T9SS) is a large multi-protein transenvelope complex distributed into the *Bacteroidetes* phylum and responsible for the secretion of proteins involved in pathogenesis, carbohydrate utilization or gliding motility. In *Porphyromonas gingivalis*, the two-component system PorY sensor and response regulator PorX participate to T9SS gene regulation. Here, we present the crystal structure of PorX_Fj_, the *Flavobacterium johnsoniae* PorX homolog. As for PorX, the PorX_Fj_ structure is comprised of a CheY-like N-terminal domain and an alkaline phosphatase-like C-terminal domain separated by a three-helix bundle central domain. While not activated and monomeric in solution, PorX_Fj_ crystallized as a dimer identical to active PorX. The CheY-like domain of PorX_Fj_ is in an active-like conformation, and PorX_Fj_ possesses phosphodiesterase activity, in agreement with the observation that the active site of its phosphatase-like domain is highly conserved with PorX.

## Introduction

The type IX secretion system (T9SS) is a nanomachine responsible for the delivery of effectors in the medium or at the cell surface, exclusively present in the *Bacteroidetes* phylum^1^. The T9SS is comprised of an outer membrane associated ring made of the PorK and PorN proteins, associated to 18 PorL–PorM inner membrane motors that extend through the periplasm to form a birdcage-like structure^2–4^. The PorL–PorM motor is proposed to provide the energy necessary for effector secretion^1,5,6^.The PorKN ring is also connected to 8 Sov/SprA translocons that are involved in the transport of effectors through the OM^4,7,8^. The T9SS secretes a broad variety of effectors including virulence factors, adhesins involved in gliding motility, S-layer proteins, and carbohydrate hydrolyzing enzymes^9^. Studies on the T9SS have been mainly performed in *Porphyromonas gingivalis* and *Flavobacterium johnsoniae*. *P. gingivalis,* an anaerobic, non-motile Gram-negative bacterium, is the major human oral pathogen associated to periodontal diseases^10,11^. Chronic *P. gingivalis* infection is also linked to rheumatoid arthritis, heart disease, diabetes, Alzheimer and other systemic diseases^12–17^. The main virulence factors involved in *P. gingivalis* pathogenicity are secreted through the T9SS. Among them, gingipains are cysteine proteases that are covalently linked to the cell surface or delivered to the extracellular milieu, and that are responsible for tissue colonization and destruction, as well as for host defense perturbation^18^. *F. johnsoniae* is an aerobic, non-pathogen bacterium living in soil and fresh water that can move along surfaces at speeds of up to 5 μm s^−1^ in a process known as gliding motility^19^. In *F. johnsoniae*, the T9SS is mainly responsible for the secretion of the SprB and RemA adhesins required for gliding motility^20^. In addition to its role in effector secretion, the T9SS GldL–GldM inner membrane complex functions as a rotary motor powering *F. johnsoniae* adhesin motion and hence gliding motility^5,6,21,22^.

While structural and functional data on the T9SS from both bacterial models accumulate, only information on *P. gingivalis* T9SS regulation are available^23–26^. A comparative genomic study first showed that the *porX* and *porY* genes co-occur with genes encoding the type IX secretion apparatus^23^. It was further shown that PorY and PorX act as cognate sensor histidine kinase and response regulator, respectively, of a two-component system (TCS)^23,24^. TCS is one of the most common signal transduction mechanisms in bacteria to sense and respond to environmental cues^27–30^. TCS is usually constituted of a histidine kinase that autophosphorylates upon a specific stimulus, and consequently transfers the phosphoryl group to the receiver domain (RD) of the response regulator. Once activated, the response regulator elicits the cellular response through its effector domain. This effector domain usually functions as a DNA-binding transcription factor, but can also display RNA-binding, protein-binding or even enzymatic activities^31^.

In contrast to the majority of TCSs, in which the components are encoded within the same operon, the *porX* and *porY* genes distribute at separate loci within the *P. gingivalis* chromosome. Nevertheless, disruption of the PorXY TCS results in the dysfunction of the T9SS, which manifests as the impaired processing of gingipains, as well as in the down-regulation of essential T9SS component genes such as *porT*, *sov*, *porKLMN*, and* porP*^23^. It was shown that PorX and PorY interact with each other^24,25^, and that PorX interacts with the cytoplasmic domain of the T9SS rotary core component PorL^24^. PorX–PorL interaction requires a hydrophobic patch at the PorL C terminus^24^, a situation that is reminiscent of the association of CheY response regulator to the flagellar C-ring FliN protein^32^. It has been therefore proposed that PorX translates the regulatory signal into a mechanical output^24^. However, the mode of action of PorX is controversial. Studies showed that PorX does not directly bind to the promoter regions of the T9SS genes^24,25^ but instead interacts with SigP, a putative extracytoplasmic function (ECF) sigma factor that itself binds to the promoter regions of the *porT*, *porV* and *porP* genes^25^. In contrast, a recent study detected a direct interaction of PorX on two DNA sequences in the *porT* gene^26^. Sequence analysis of PorX showed that it is constituted of an N-terminal RD of the CheY family and a C-terminal alkaline phosphatase-like domain of the PglZ family separated by a linker region^24^. The C-terminal domain is not involved in T9SS gene regulation, and the linker region was proposed to interact with the PorX DNA target^26^. Very recently, a study provided further insights onto the structure–function relationship of PorX. It was notably shown that the PorX PglZ domain possesses a phosphodiesterase activity, and that dimerization of PorX, promoted by the presence of zinc, is required for its activity^33^. Substrates for the PglZ domain were determined, and an interdependence between the RD and PglZ domains through the dimerization surface was established. Furthermore, the crystal structures of the dimeric, activated PorX, as well as its complex with pGpG, were solved^33^.

Until now, there is no information on T9SS regulation by a TCS in *F. johnsoniae*. However, PorX homologs are present in all species encoding T9SS components^34^, arguing that PorX is indispensable for the regulation of T9SS activity. In this study we produced and purified the *F. johnsoniae* PorX homolog (*Fjoh_2906*, called hereafter PorX_Fj_) and solved its structure by X-ray crystallography at 2.0 Å resolution. Comparison with the *P. gingivalis* PorX (PorX_Pg_) structure suggests that both proteins share similar function. Indeed, we confirm that PorX_Fj_ has phosphodiesterase activity and further show that PorX_Fj_ interacts with the cytoplasmic domain of the PorL homologue GldL, and that this interaction is mediated by the phosphorylated CheY-like domain of PorX_Fj_.

## Results

### Overall structure of PorX_Fj_

In order to solve the structure of PorX_Fj_, the native protein as well as its Selenomethionine-labeled (SeMet) derivative were produced and purified by immobilized ion metal affinity and size-exclusion chromatographies. Diffracting crystals of both proteins grew in the P2_1_2_1_2_1_ space group; the SeMet PorX_Fj_ structure was solved using a SAD data set and then used as a model to solve a higher resolution structure of the native PorX_Fj_ by molecular replacement.

**Figure 1:**
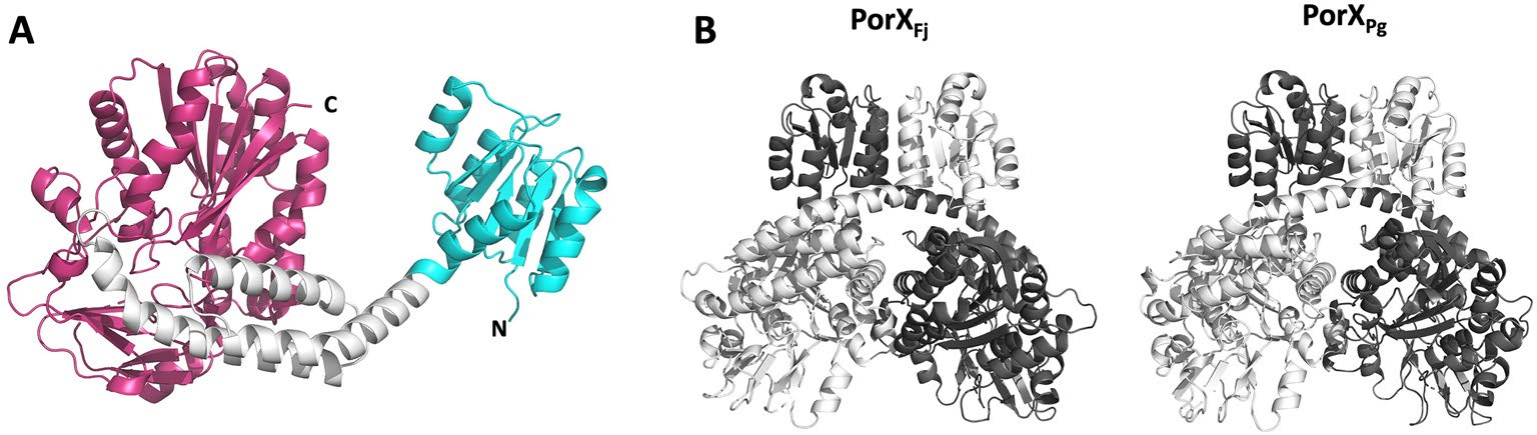
Overall structure of PorX_Fj_. (**A**) Structure of the PorX_Fj_ monomer ; the CheY-like N-terminal, the HBD and the PglZ C-terminal domains are shown in cyan, white and purple, respectively; the N- and C-termini are labelled. (**B**) Comparison of the PorX_Fj_ (left) and PorX_Pg_ (right) dimer structures; For each structure, molecules A and B are shown in light and dark grey, respectively.

Similarly to the recently released *P. gingivalis* PorX_Pg_ structure^33^, PorX_Fj_ displays three distinct domains: a N-terminal CheY-like RD (residues 2–121), followed by a three-helix bundle domain (HBD, residues 122–205), and a C-terminal PglZ domain (residues 206–517) (Fig. 1A). Two PorX_Fj_ molecules are present in the asymmetric unit. While the three domains of each molecule are perfectly superimposable independently (with an rmsd of 0.24 Å, 0.54 Å and 0.22 Å for the N-terminal domain, HBD and C-terminal domain, respectively), superposition of the two whole molecules yields an rmsd of 1.47 Å. Indeed, when the two molecules are superimposed through their N- or C-terminal domains, a slight shift of the HBD's first helix is observed, which carries over to the rest of the molecule and could reflect some flexibility on either side of the kink present in this helix (Fig. S1).

The two PorX_Fj_ molecules form an intertwined dimer identical to the PorX_Pg_ dimer^33^, with a buried surface area of 2985 Å^2^ (representing 12.5% of the total surface area) and a binding energy of − 42.7 kcal mol^−1^, according to QtPISA^35^ (Fig. 1B). The dimer is stabilized by a network of electrostatic interactions (21 hydrogen bonds and 6 salt bridges), as well as hydrophobic patches (involving 21 hydrophobic residues from each monomer), principally between the N- and C-terminal domain of each monomer (Fig. S2; Table S1).

**Figure 2:**
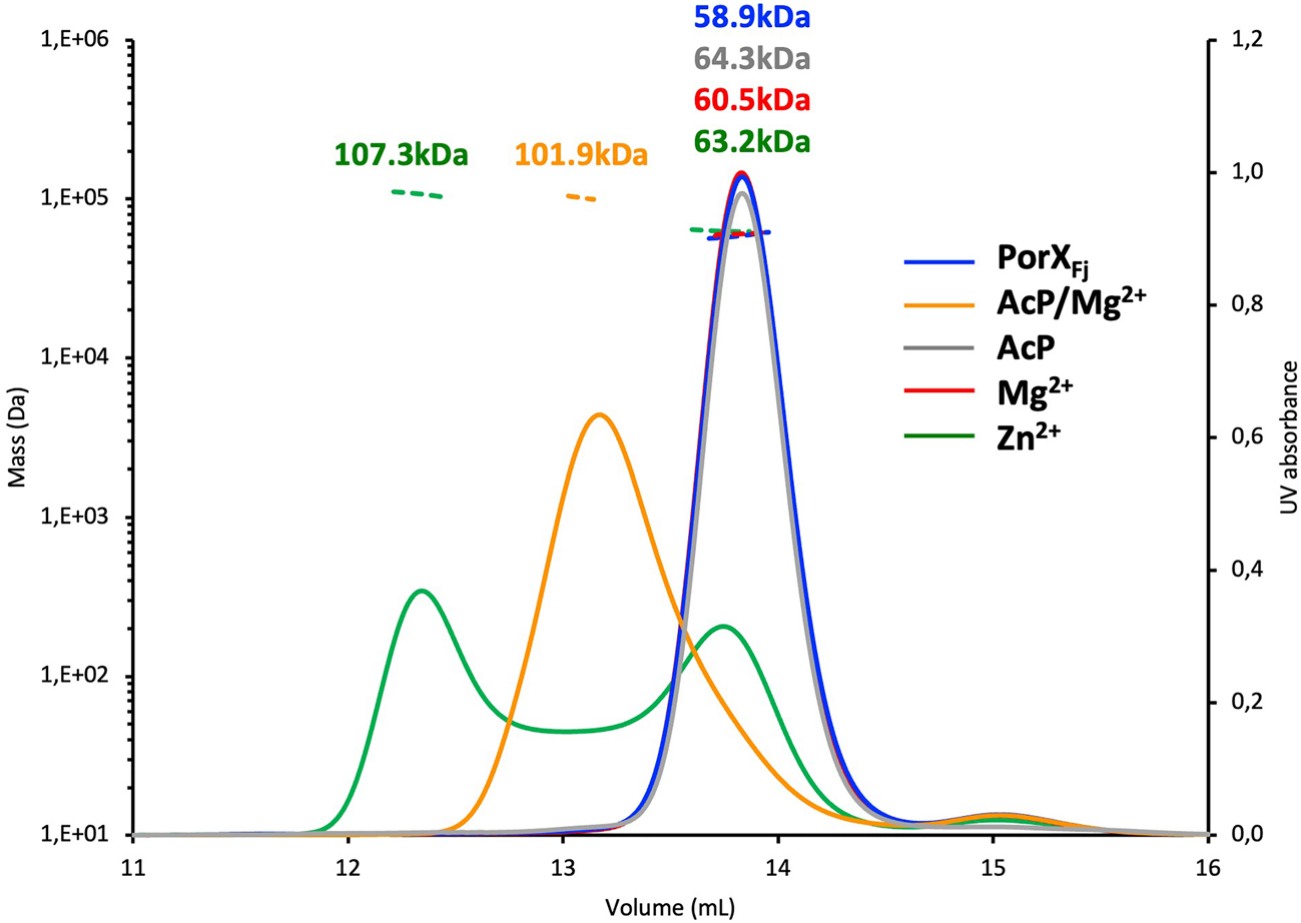
PorX_Fj_ dimerization is induced by phosphorylation and Zn^2+^. The purified PorX_Fj_ alone or incubated with AcP and MgCl_2_ (AcP/Mg^2+^), AcP (AcP), MgCl_2_ (Mg^2+^) or ZnCl_2_ (Zn^2+^) was analyzed by size-exclusion chromatography-multiangle light scattering (SEC-MALS). Note that the chromatograms of PorX_Fj_ alone and incubated with MgCl_2_ overlap and are hence almost indistinguishable.

Similarly to PorX_Pg_, PorX_Fj_ is monomeric in solution and dimerization is induced by phosphorylation with the low-molecular weight phospho-donor acetyl phosphate (AcP) in presence of Mg^2+^, but not by AcP nor Mg^2+^ alone, as shown by SEC-MALS results (Fig. 2). No crystal of monomeric PorX_Pg_ could be obtained, and its dimeric structure was solved after incubation with BeF_3_, a compound used to mimic phosphorylation^33^. As no BeF_3_ was added to PorX_Fj_, we hypothetize that formation of the dimer was promoted by the crystallization process. Noteworthy, 41 residues have their side chains involved in the stabilization of the PorX_Fj_ dimer through electrostatic interactions and hydrophobic contacts, compared to the 29 residues in the PorX_Pg_ dimer (Fig. S3). This difference suggests that the PorX_Fj_ dimer is more stable and could be more prone to assemble during crystallization.

### The CheY-like domain

**Figure 3:**
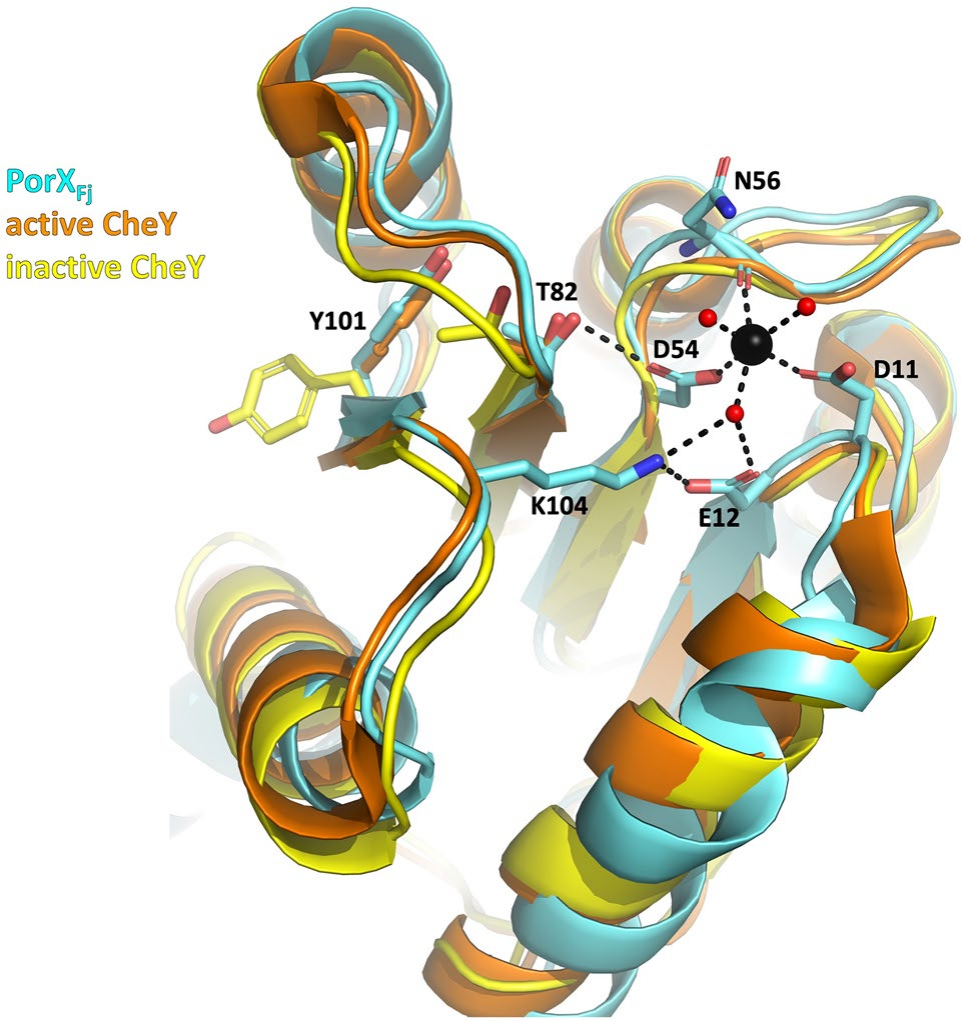
The PorX_Fj_ CheY-like N-terminal domain. Overlay of the PorX_Fj_ (cyan), active CheY (orange; PDB: 1FQW) and inactive CheY (yellow; PDB: 2CHE) structures. For clarity purpose, only the side chains of the CheY ‘switch’ residues (corresponding to PorX_Fj_ T82 and Y101) are displayed. The Mg^2+^ ion and the water molecules are shown as black and red spheres, respectively, and the hydrogen bonds are indicated by dashed black lines.

The CheY-like RD adopts the classical (α/β)_5_ doubly-wound fold consisting of a central 5-stranded β-sheet, surrounded by five α-helices. The conserved active site is an acidic pocket formed by three acidic residues (D11, E12 and D54), T82, and K104. PorX_Fj_ was not activated by phosphorylation before crystallization, and indeed no phosphoryl group attached to the conserved D54 residue is present in the electron density map. The putative binding site of the metal supposed to stabilize the phosphoryl group is clearly occupied by an ion that is octahedrally coordinated by the carboxylate oxygens of D11 and D54, the main chain-carbonyl oxygen of N56 and three water molecules (Fig. 3). The nature of the ion cannot be attributed unambiguously from inspection of the electron density. By comparison with the PorX_Pg_ structure, and accordingly to the coordination number and the bond lengths between the ion and its ligands, a Mg^2+^ ion was modelled in the structure. As no magnesium was added during the purification and crystallization steps, we can therefore speculate that the ion present in the structure was acquired intracellularly. Despite the absence of BeF_3_ that mimics the activating phosphorylation of the RD, the side chains of the two PorX_Fj_ highly conserved ‘switch’ residues T82 and Y101 are oriented towards the active site, in a conformation corresponding to the active state of RDs (Fig. 3). This confirms that binding of the Mg^2+^ ion is likely sufficient to induce the conformational changes associated to RDs activation, while phosphorylation stabilizes this Mg^2+^-bound conformation, as previously proposed^36^.

### The HBD domain

The PorX_Fj_ HBD folds as a three-helix bundle, and presents the same features as in the *P. gingivalis* PorX_Pg_ structure: a 80° bend of the polypeptide chain at its N terminus, and a kink at the end of the first helix. In PorX_Pg_, this domain was proposed to interact with DNA^26^. However, the first hits returned by a structural similarity search within the Protein Data Bank (PDB) using the DALI server^37^ correspond to domains involved in protein–protein interaction: the fibronectin-binding protein RevA^38^, the plakin domain of plectin^39^, or the BAG domain^40^ for instance.

### The PglZ domain

**Figure 4:**
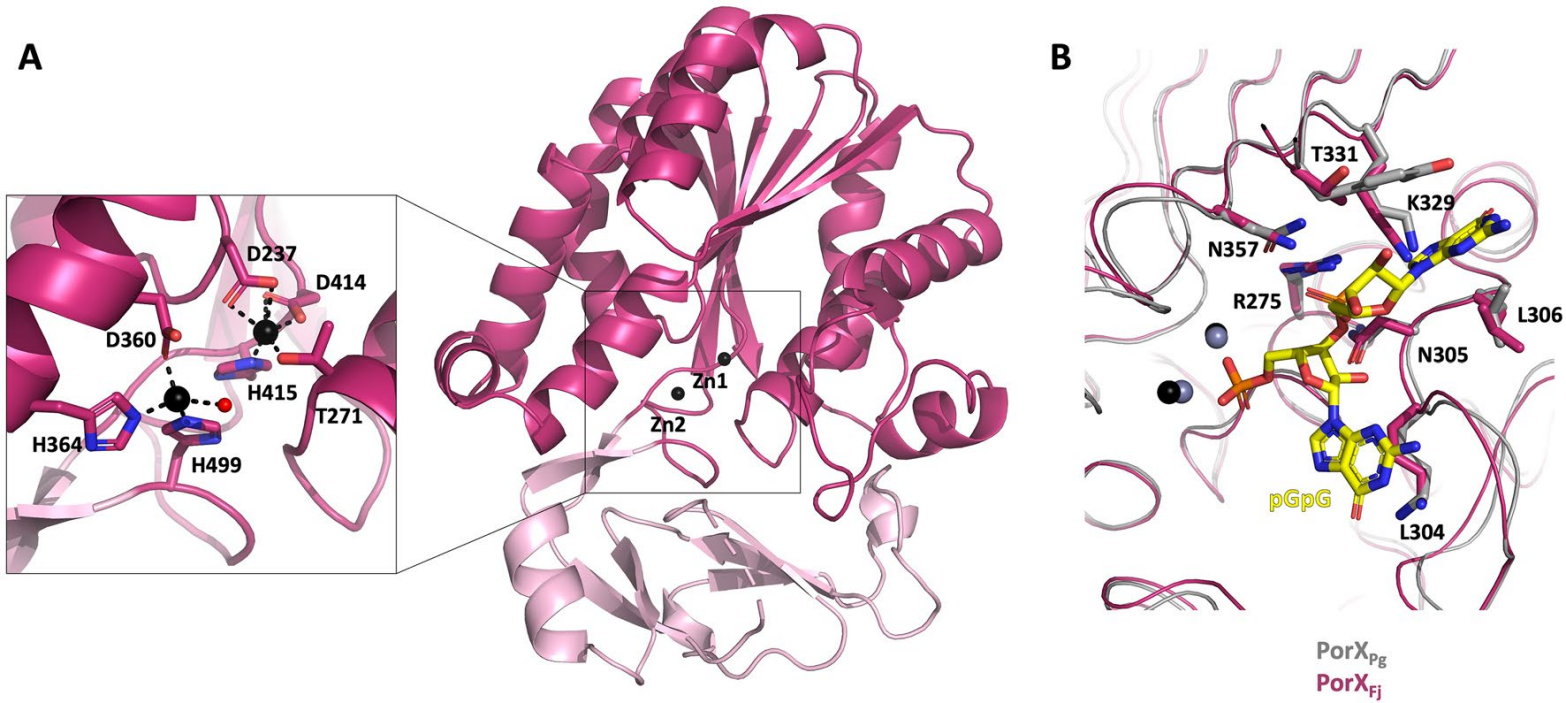
The PorX_Fj_ PglZ C-terminal domain. (**A**) The catalytic and cap subdomains are shown in magenta and pink, respectively. The two Zn^2+^ ions (Zn1 and Zn2) are indicated as black spheres. Enclosed is a close-up view of the Zn^2+^ ions coordination, with the water molecule and the hydrogen bonds displayed as a red sphere and dashed black lines, respectively. (**B**) Overlay of the ligand-binding region of PorX_Fj_ (magenta) with PorX_Pg_ (grey) in complex with pGpG (yellow). The Zn^2+^ ions present in PorX_Fj_ and PorX_Pg_ are shown as black and grey spheres, respectively. The side chains of PorX_Pg_ residues in contact with pGpG and of the corresponding PorX_Fj_ residues are indicated. For clarity purpose, only PorX_Fj_ residues are labelled, and the molecules are slightly rotated compared to panel (**A**).

The PorX_Fj_ C-terminal PglZ domain adopts the classical α–β–α fold of the alkaline phosphatase superfamily, which consists of a catalytic domain with a central β-sheet of six β-strands surrounded by six and four α-helices on each side, and a β-rich cap subdomain at the entrance of the active site (Fig. 4A). The active site coordinates two Zn^2+^ ions: Zn1 is coordinated by the catalytic T271, D237, D414 and H415 residues while Zn2 is coordinated by residues H364, H499, D360 and a water molecule (Fig. 4A). Of note, the distance between the two ions is 5.4 Å, a distance much greater than the distance reported for any other alkaline phosphatase superfamily members^41^. In the PorX_Pg_ dimer, each monomer presents a distinct conformation of residues 359–367: in one monomer this region folds as an α-helix (conformation HH), while in the other it folds as an extended loop (conformation HL)^33^. As the HL conformation is not compatible with Zn2 binding, the monomer with this conformation is unlikely to be active. In contrast, the two monomers of the PorX_Fj_ dimer adopt the HH conformation with two coordinated Zn^2+^ ions and are therefore likely to be active. Comparison with the structure of an inactive PorX_Pg_ mutant (T272A) crystallized in complex with pGpG reveals that residues interacting with the ligand are strictly conserved in PorX_Fj_, except Y332 that is replaced by T331 (Fig. 4B). However, the electron density around Y332 and its contacting pGpG purine group is poorly defined^33^, suggesting that this interaction is labile and could be not critical for the catalyzed reaction.

### Purified PorX_FJ_ has phosphodiesterase activity

**Figure 5:**
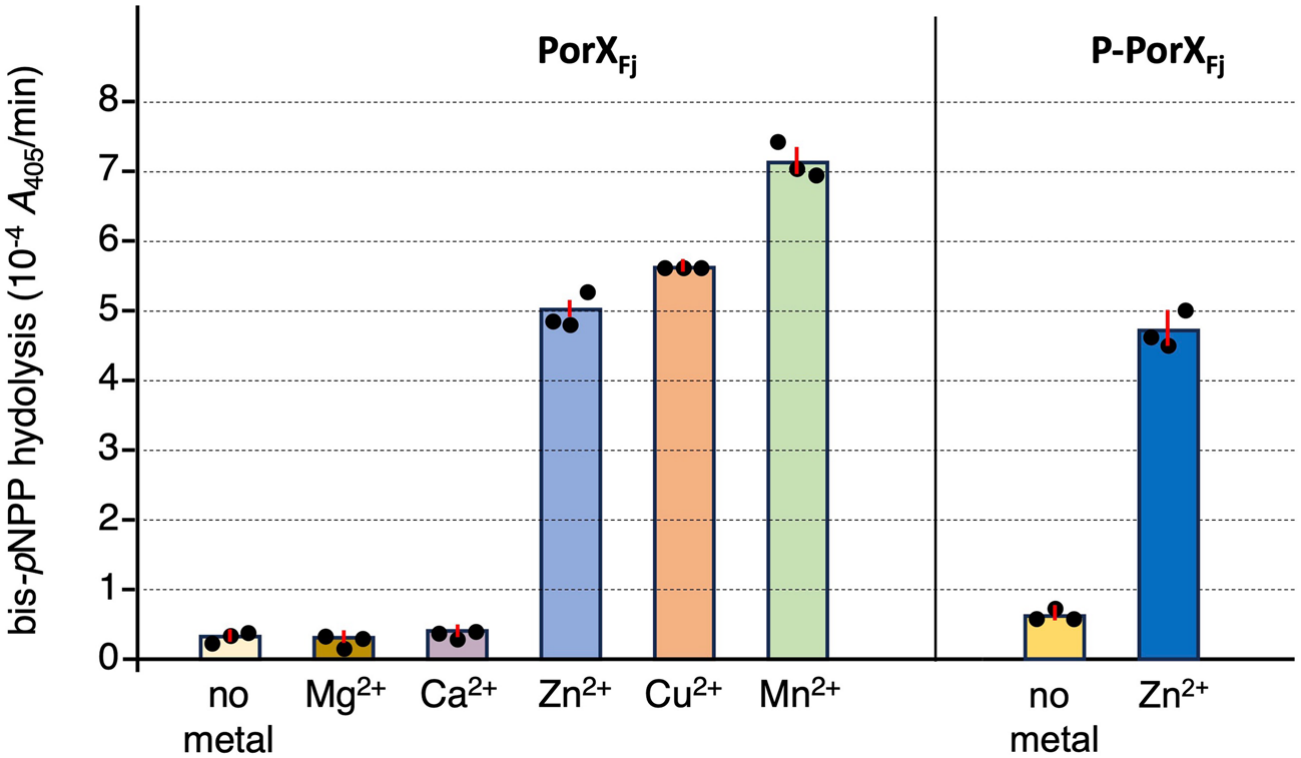
PorX_Fj_ has metal-dependent phosphodiesterase activity. The phosphodiesterase activity of PorX_Fj_ or phosphorylated PorX_Fj_ (pre-incubated with acetylphosphate and MgCl_2_, P-PorX_Fj_) was measured using bis-*p*-nitrophenyl phosphate (bis-*p*NPP) as substrate. The average activity (nitrophenol released (measured at *A*_405_) per minute, from three independent measurements) is represented as a bar, with the three raw values (closed circles) and standard deviation (red vertical line). Reaction buffer was supplemented with MgCl_2_ (Mg^2+^), CaCl_2_ (Ca^2+^), CuCl_2_ (Cu^2+^), ZnCl_2_ (Zn^2+^), MnCl_2_ (Mn^2+^) or no metal.

PorX_Pg_ was previously shown to have phosphodiesterase activity in vitro^33^. Enzymatic assays using the purified PorX_Fj_ protein showed that it also hydrolyzes bis-*p-*nitrophenyl-phosphate (Fig. 5). Similarly to PorX_Pg_, this activity requires the presence of a divalent cation, but unlike PorX_Pg_, PorX_Fj_ is active not only in presence of Zn^2+^, but also in presence of Cu^2+^ and Mn^2+^ (Fig. 5). The presence of the divalent cation is necessary but also sufficient for PorX_Fj_ activity as a comparable activity was measured when the protein was phosphorylated or not prior to the reaction (Fig. 5). Such a broad metal specifity was also observed for the *Sinorhizobium meliloti* PhnA alkaline phosphatase^41^. However, comparison of the PorX_Pg_ and PorX_Fj_ structures highlights no significant structural difference in the ions pocket that could explain the different specificity of the two proteins.

**Figure 6:**
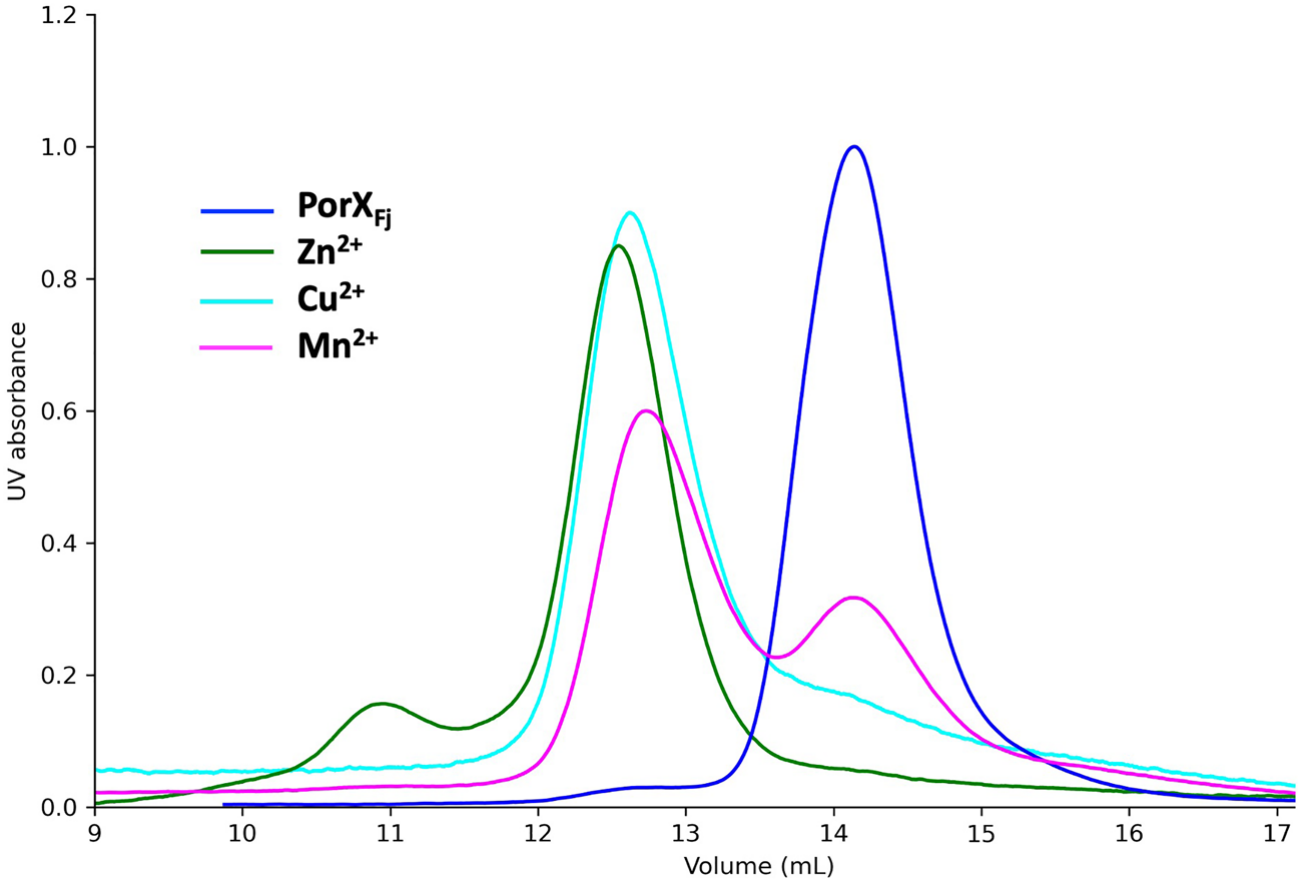
PorX_Fj_ dimerization is induced by different metals. The purified PorX_Fj_ alone or incubated with ZnCl_2_ (Zn^2+^), CuCl_2_ (Cu^2+^) or MnCl_2_ (Mn^2+^) was analyzed by size-exclusion chromatography (SEC). Note that the column was equilibrated in PBS supplemented with the corresponding metal solution.

SEC-MALS analysis showed that PorX_Fj_ incubation with Zn^2+^ induced dimerization, similarly to PorX_Pg_^33^ (Fig. 2). Interestingly, dimers induced by phosphorylation (AcP + Mg^2+^) and Zn^2+^ eluted at different volumes, suggesting that they adopt different conformations, which confirms SAXS analysis of PorX_Pg_^33^. Moreover, a dimer/monomer equilibrium was observed when PorX_Fj_ was incubated with Zn^2+^, which could be explained by a dilution effect as the SEC-MALS analysis was carried out with the column equilibrated in PBS only. Therefore, SEC analysis of PorX_Fj_ dimerization induced by the different ions necessary for activity (Zn^2+^, Cu^2+^, and Mn^2+^) was carried out with the column equilibrated with PBS supplemented with the corresponding metal solutions. As expected, full dimerization was observed in the presence of Zn^2+^ and Cu^2+^ (Fig. 6). A dimer/monomer equilibrium was still observed in the presence of Mn^2+^ that could reflect a lower affinity of PorX_Fj_ for this ion.

### The CheY domain of PorX_Fj_ mediates interaction with GldL

**Figure 7:**
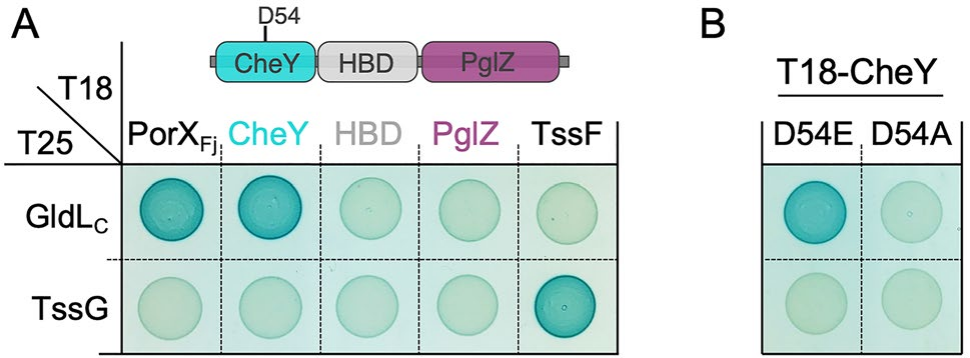
The activated form of the PorX_Fj_ CheY-like domain interacts with GldL. Bacterial two-hybrid assay. BTH101 reporter cells producing the indicated domains/proteins (**A**, same color code as in Fig. 1: CheY domain, cyan; HBD, grey; PglZ domain, magenta) and CheY D54 phosphomimetic (D54E) and phosphoablative (D54A) variants (**B**) fused to the T18 and T25 domain of the *Bordetella* adenylate cyclase were spotted on X-Gal-IPTG reporter LB agar plates. The blue color of the colony reports interaction between the two partners. Controls include T18 and T25 fusions to TssF and TssG, two T6SS proteins from enteroaggregative *E. coli* that interact but unrelated to the T9SS^43^.

The PorX_Pg_ protein was shown to interact with PorL^24^, a component of the PorLM rotor^5,6,21,42^, a situation resembling the interaction of CheY with the FliN subunit of the flagellar C-ring. Bacterial two-hybrid (BACTH) assays showed that this is also the case of PorX_Fj_, which interacts with the cytoplasmic domain of the PorL homologue, GldL (GldL_C_, Fig. 7A). Interestingly, reminiscent of the CheY/FliN interaction, our analyses defined that the PorX_Fj_/GldL_C_ interaction is mediated by the PorX_Fj_ CheY-like RD (Fig. 7A). We further tested whether activation of the CheY-like domain of PorX_Fj_ is required for GldL interaction. Figure 7B shows that while a phosphomimetic substitution of the phosphorylated D54 residue (D54E) maintains the interaction with GldL, a phosphoablative substitution (D54A) prevents PorX_Fj_–GldL interaction.

## Discussion

In *P. gingivalis*, the T9SS activity was shown to be regulated in part by the TCS composed of the histidine sensor PorY and the response regulator PorX_Pg_. The mode of action of PorX_Pg_ remains elusive, notably on how its phosphodiesterase activity and its phosphorylation status influence T9SS activity. In addition, its potential interaction with DNA is controversial. It was recently shown that PorX_Pg_ activation is induced by phosphorylation of its RD, resulting in dimerization of the protein, and the structure of active, dimeric PorX_Pg_ was solved^33^. In this study, we solved the crystal structure of PorX_Fj_, the *F. johnsoniae* PorX homolog. Comparison with the PorX_Pg_ structure strongly suggests that the two proteins share similar functions. Indeed, PorX_Fj_ possesses phosphodiesterase activity, it crystallized as a dimer identical to the active PorX_Pg_ dimer, the CheY-like RD adopts an active-like conformation, and the active site of the PglZ phosphatase-like domain is highly conserved with PorX_Pg_. Interestingly, crystallization is sufficient to promote dimerization of PorX_Fj_ without prior activation by phosphorylation, as previously observed^44^. We propose that PorX_Fj_ dimerization relies on the binding of the Mg^2+^ ion to the PorX_Fj_ RD, inducing its active-like conformation, in conjunction with high concentration conditions occurring during the crystallization process that promote molecular contacts. PorX_Pg_ dimerization was not observed in the absence of activation, probably due to a less stable dimer than PorX_Fj_. Unfortunately, there is no monomeric PorX structure that could provide clues to the dimerization process, particularly if conformational changes are involved.

Similarly to PorX_Pg_, PorX_Fj_ lacks any canonical DNA-binding motif, which further questions about the ability of PorX, or PorX_Fj_, to directly interact with DNA. Rather, PorX_Pg_ interacts with the extracytoplasmic function (ECF) sigma factor SigP, that itself interacts with DNA^25^. Only one homolog of SigP is present in *F. johnsoniae* (GenBank: PZQ89414.1). Further investigation would be necessary to analyze the putative interaction of PorX_Fj_ with the SigP homolog, and more generally to assess its involvement in T9SS gene regulation. In addition to its interaction with SigP, PorX_Pg_ was shown to interact with the cytoplasmic domain of PorL, a component of the PorLM motor that uses the proton-motive force (PMF) to energize effector secretion through the T9SS^5,6,24,45^. Our results demonstrated that this interaction is conserved in *F. johnsioniae*, suggesting that PorX_Fj_ may regulate T9SS activity. In addition, we showed here that the PorX_Fj_ CheY-like domain is sufficient to mediate interaction with the PorL homolog, GldL. The interaction of a CheY-like protein with a multiprotein complex is well documented in the case of the regulation of the flagellum rotation, in which the chemotaxis CheY protein interacts with FliM and FliN, two components of the C-ring^32,46^. Binding of phosphorylated CheY to FliM/N induces a tilting movement of the C-ring that repercutates onto FliG to reverse the direction of motor rotation^32,47–51^. Interestingly, mutagenesis of the conserved phosphorylable aspartate residue D54 further showed that a substitution preventing D54 phosphorylation prevents PorX_Fj_ binding to GldL. Taken together, we propose that the PorX_Fj_ phosphorylation status controls association to and dissociation from the GldLM complex. We further speculate that this interaction regulates the activity of the motor as a response of an environmental cues or of PglZ domain activity. In the case of the flagellum, CheY association to the C-ring controls the reversible switch between clockwise and counterclockwise rotation, hence enabling cells to swim towards favorable chemical habitats^50,51^. In *F. johnsioniae*, the PMF-dependent activity of the T9SS GldLM rotor powers effector transport through the outer membrane but also the movement of the SprB adhesin at the cell surface, hence supporting gliding^5,6,45,52^. One may hypothesize that PorX_Fj_ may have a function comparable to CheY by switching the T9SS motor to different conformations allowing to control effector secretion or adhesin displacement. PorX_Fj_ may thus control the speed of gliding or the gliding direction. Further studies should be performed to better understand the contribution of PorX_Fj_, and of its CheY, HBD and PglZ domain in gliding motility, T9SS gene regulation and SigP interaction.

## Experimental procedures

### Bacterial strains, media, chemicals and growth conditions

*Escherichia coli* K-12 strain DH5a was used for all cloning procedures, T7 strain for protein production, and BTH101 for bacterial two-hybrid assays, *E. coli* cells were grown in Lysogeny Broth (LB) or Turbo broth supplemented with antibiotics when necessary (kanamycin 50 µg mL^−1^, ampicillin 100 µg mL^−1^). Expression from pLIC03 and BACTH vectors was induced with 1 mM and 0.5 mM of isopropyl β-d-1-thiogalactopyranoside (IPTG), respectively.

### Plasmid construction

The sequence encoding full length PorX_Fj_ (*Fjoh_2906*) was amplified from *F. johnsoniae* genomic DNA (ATCC17061/DSMZ2064) using the following primers: 5ʹ-CCTGTACTTCCAATCAATGGATAAGATAAGAATACTTTGGGTCG and 5ʹ-CCGTATCCACCTTTACTTTATTATTTAGGGTTAAATACCAAAAACGG. The sequence was cloned into pLIC03 (kindly provided by the BioXtal company, Marseille) using the In-Fusion technology (Takara), following the manufacturer protocol. The pLIC03 expression vector is a pET-28a + derivative (Novagen) carrying a cassette coding for a His_6_ tag and a Tobacco Etch Virus (TEV) protease-cleavage.

BACTH vectors producing TssF, TssG and GldL_C_ fused to the *Bordetella* adenylate cyclase T18 or T25 domains have been previously published^6,43^. BACTH plasmid producing PorX_Fj_ and PorX_Fj_ domains fused to the T18 and T25 domains were constructed by restriction free (RF) cloning^53^ using oligonucleotides *CGCCACTGCAGGGATTATAAAGATGACGATGACAAG*GATAAGATAAGAATACTTTGGGTCGATGATGAG and *CGAGGTCGACGGTATCGATAAGCTTGATATCGAATTCTAG*TTATTTAGGGTTAAATACCAAAAACGGAATAATCATTTC (T18-PorX_Fj_), *CGCCACTGCAGGGATTATAAAGATGACGATGACAAG*GATAAGATAAGAATACTTTGGGTCGATGATGAG and *CGAGGTCGACGGTATCGATAAGCTTGATATCGAATTCTAG*TTATTTTTGGTAATCTAATGTTGTTTTTTCTGTAATCAGTC (T18-CheY), *CGCCACTGCAGGGATTATAAAGATGACGATGACAAG*GAATTCCGCAAAATCTCGATGGAATTAGC and *CGAGGTCGACGGTATCGATAAGCTTGATATCGAATTCTAG*TTATTTTGGAGCAAACCAGTCTTCGTAATTTC (T18-HBD), and *CGCCACTGCAGGGATTATAAAGATGACGATGACAAG*GCAGATAAACCAATTCAATCTCATAATTTATTTAAAGAATTAGTTG and *CGAGGTCGACGGTATCGATAAGCTTGATATCGAATTCTAG*TTATTTAGGGTTAAATACCAAAAACGGAATAATCATTTC (T18-PglZ) (sequences annealing on the target plasmids in italics). Briefly, the DNA fragment was amplified using primers that introduced extensions annealing to the target vector. The double-stranded product of the first PCR has then been used as primer for a second PCR using the target vector as template. PCR products were then treated with DpnI to eliminate template plasmids and transformed into DH5a-competent cells. Substitutions were introduced by site-directed mutagenesis using complementary oligonucleotides bearing the desired mutation (CTTTGACATTGTTTTTCTT*GCC*GAAAATATGCCGGGAATG and CATTCCCGGCATATTTTC*GGC*AAGAAAAACAATGTCAAAG for D54A, CTTTGACATTGTTTTTCTT*GAG*GAAAATATGCCGGGAATG and CATTCCCGGCATATTTTC*CTC*AAGAAAAACAATGTCAAAG for D54E; mutagenized codon in italics, mutagenized bases underlined). All plasmids have been verified by DNA sequencing (Eurofins).

### Protein production, purification and analysis

PorX_Fj_ was produced in *E. coli* T7 cells cultured in Turbo Broth medium at 37 °C. At OD_600nm_ of 0.6–0.8, *porX*_*Fj*_ expression was induced by adding 1 mM IPTG and the bacterial growth was pursued for 18 h at 17 °C. Cells were harvested by centrifugation at 4000×*g* for 10 min, resuspended in lysis buffer (50 mM Tris–HCl pH 8.0, 300 mM NaCl, 10 mM imidazole, 250 µg mL^−1^ lysozyme, 1 mM PMSF) and frozen overnight at − 20 °C. After thawing, 20 µg mL^−1^ of DNase and 1 mM of MgSO_4_ were added, and cells were lysed by sonication. The pellet and soluble fractions were separated by centrifugation at 16,000×*g* for 30 min, and the His_6_-tagged protein was purified from the soluble fraction by immobilized metal ion affinity chromatography using a 5 mL HisTrap crude (GE Healthcare) Ni^2+^-chelating column equilibrated in buffer A (50 mM Tris–HCl p H8.0, 300 mM NaCl, 10 mM imidazole). The protein was eluted with buffer A supplemented with 250 mM imidazole and further purified by size exclusion chromatography (HiLoad 16/60 Superdex 200 prep grade, GE Healthcare) equilibrated in 10 mM HEPES pH 7.5, 500 mM NaCl. Selenomethionine-labeled (SeMet) PorX_Fj_ was produced and purified with the same protocol as the native PorX_Fj_, except that the cells were grown in SeMet minimal medium^54^.

### Dimerization analysis

For the Size-Exclusion Chromatography Multi-Angle Light Scattering (SEC-MALS) analysis, the purified PorX_Fj_ (1.6 mg mL^−1^) was incubated for 1 h at RT with 20 mM acetyl phosphate (AcP) + 10 mM MgCl_2_, or 20 mM AcP, or 10 mM MgCl_2_, or 100 µM ZnCl_2_. The samples were loaded on a Superdex 200 Increase 10/300 GL column (GE Healthcare) equilibrated in PBS at a flow rate of 0.6 mL min^−1^, using an Ultimate 3000 HPLC system (Fischer Scientific). Detection was performed using an eight-angle light-scattering detector (DAWN8, Wyatt Technology) and a differential refractometer (Optilab, Wyatt Technology).

For the Size-Exclusion Chromatography (SEC) analysis, the purified PorX_Fj_ (1.6 mg mL^−1^) was incubated for 1 h at RT with 100 µM ZnCl_2_, MnCl_2_, or CuCl_2_. The samples were loaded on a Superdex 200 Increase 10/300 GL column (GE Healthcare) equilibrated in PBS supplemented with100 µM of the corresponding metal solution.

### Crystallization, data collection and processing

The purified PorX_Fj_ and SeMet PorX_Fj_ were concentrated to 10 and 12 mg mL^−1^, respectively. Crystallization trials were performed using the sitting-drop vapor-diffusion method at 293 K in 96-well Swissci-3 plates, with Stura Footprint (Molecular Dimensions), Wizard I and II (Rigaku), Structure I and II (Molecular Dimensions) and JCSG + (Qiagen) screens, and using Tecan and Mosquito (TTP Labtech) robots to fill in the plates and dispense the drops, respectively. PorX_Fj_ crystals appeared in condition No. 20 from Structure I screen (0.2 M calcium acetate, 0.1 M sodium cacodylate pH 6.5, 18% PEG 8000). SeMet PorX_Fj_ crystals appeared in several condition, and after optimization^55^, the final crystallization conditions were 0.2 M ammonium sulfate, 0.05 M sodium acetate, 0.05 M sodium citrate pH 5.0–6.0, 10–30% PEG 2000. Crystals were mounted in cryo-loops (Hampton CrystalCap Magnetic) and were briefly soaked in crystallization solution supplemented with 20% (v/v) polyethylene glycol. The crystals were flash-cooled in a nitrogen-gas stream at 100 K using a home cryocooling device (Oxford Cryosystems).

Native diffraction data of PorX_Fj_ and single wavelength anomalous dispersion (SAD) data of SeMet PorX_Fj_ were collected to 2.0 Å and 2.3 Å resolution, respectively, on beamline Proxima-1 at the Soleil synchrotron (Paris, France). The data sets were integrated with XDS and scaled with SCALA^56^ from the CCP4 suite^57^. Heavy atom substructure determination of SeMet PorX_Fj_, phase calculations and density modification were performed using HySS^58^, Phaser^59^ and Parrot^60^, respectively, as implemented in the Phaser SAD pipeline from the CCP4 suite. A partial SeMet PorX_Fj_ model was built automatically in Buccaneer^61^, completed manually in COOT^62^, and was subsequently used as model for molecular replacement with MOLREP^63^ to solve the structure of native PorX_Fj_. Refinement, correction, and validation of the structure were performed with autoBUSTER^64^, COOT, and Molprobity^65^, respectively. Data collection and refinement statistics are reported in Table 1.

**Table 1:** Data collection and refinement statistics of PorX_Fj_.

	PorX_Fj_	SeMet PorX_Fj_
Data collection		
Space group	P2_1_2_1_2_1_	P2_1_2_1_2_1_
*a*, *b*, *c* (Å)	84.6, 97.5, 131.1	83.0, 100.0, 129.3
α, β, γ (°)	90, 90, 90	90, 90, 90
Resolution (Å)*	40–2.0 (2.11–2.0)	30–2.3 (2.42–2.3)
Unique reflections*	73,936 (10,652)	48,780 (6957)
Redundancy*	6.7 (7.0)	13.6 (13.8)
Completeness (%)*	100.0 (100.0)	99.8 (99.0)
I/σ*	8.1 (0.6)	17.0 (1.5)
R_meas_ (%)*	12.4 (226.2)	8.3 (161.1)
CC1/2	0.998 (0.319)	0.999 (0.604)
Mosaicity (°)	0.14	0.12
Refinement and model quality		
Resolution (Å)*	30.0–2.0 (2.01–2.0)	
Reflections*	73,840 (1396)	
R_fac_/R_free_ (%)	21.6/25.5	
N° atoms: protein/ligand/ion/water	8398/24/6/560	
B-factors (Å^2^): protein/ligand/ion/water	53.5/81.6/41.8/57.3	
Rmsd: bond (Å)/angle (°)	0.008/0.98	
Ramachandran (%)		
Most favoured	94.7	
Allowed regions	4.6	
Outliers	0.7	
PDB accession code	8P6F	

*Values in parentheses are for the highest-resolution shell.

### Bacterial two-hybrid assays.

Bacterial two-hybrid assays were conducted as previously described^43^. The proteins or domains to be tested were fused to the isolated T18 and T25 catalytic domains of the *Bordetella* adenylate cyclase. After introduction of the two plasmids producing the fusion proteins into the BTH101 reporter strain, plates were incubated at 28 °C for 24 h. Three independent colonies for each transformation were inoculated into 600 μL of LB medium supplemented with ampicillin, kanamycin, and IPTG (0.5 mM). After overnight growth at 28 °C, 15 μL of each culture were spotted onto LB plates supplemented with ampicillin, kanamycin, IPTG, and X-Gal and incubated at 28 °C. Controls include interaction assays with TssF and TssG, two T6SS protein partners from enteroaggregative *E. coli*^43^ unrelated to the T9SS. The experiments were done at least in triplicate and a representative result is shown.

### Phosphodiesterase activity assay

PorX_Fj_ phosphodiesterase activity was measured using bis-*p*-nitrophenyl phosphate (bis-*p*NPP, Sigma-Aldrich) as substrate, essentially as previously published^33^ (Schmitz et al. 2022). Briefly, purified PorX_Fj_ was phosphorylated by incubation for 1 h in the presence of 20 mM of acetyl-phosphate and 10 mM of MgCl_2_, before being desalted on a 7-kDa Zeba™ Spin desalting column (ThermoFischer Scientific). 50 μL of 2 μM purified phosphorylated or non-phosphorylated PorX_Fj_ in 50 mM Tris–HCl pH8, 150 mM NaCl were mixed with 50 μL of 50 mM Tris–HCl pH8.5, 150 mM NaCl, 10 mM bis-*p*NPP supplemented, or not, with 100 μM of MgCl_2_, 100 μM of CaCl_2_, 100 μM of CuCl_2_, 100 μM of MnCl_2_ or 100 μM ZnCl_2_ in a 96-well microplate (transparent, flat bottom, Nunc). The release of *p*-nitrophenol was monitored for 180 min at 37 °C by measuring the absorbance at 405 nm (*A*_405_) using a TECAN microplate reader. A control experiment with bis-*p*NPP only was also performed to measure the spontaneous hydrolysis, and the values of bis-*p*NPP spontaneous hydrolysis were subtracted to the measures of PorX_Fj_ activity. Bis-*p*NPP activity was calculated from the slope and reported as *A*_405_ per minute. The experiments were done in triplicate.

### Supplementary Information

Supplementary Information.

## Data Availability

Crystallographic atomic coordinates and structure factors have been deposited in the PDB with the accession code 8P6F.
