# Supplementary material for: Structure–function analysis of PorXFj, the PorX homolog from Flavobacterium johnsioniae, suggests a role of the CheY-like domain in type IX secretion motor activity
Source: Sci Rep. 2024 Mar 19;14:6577. doi: 10.1038/s41598-024-57089-9 (PMC10951265; doi:10.1038/s41598-024-57089-9)
Supplement: Supplementary file 1 — Supplementary Information. [file 41598_2024_57089_MOESM1_ESM.pdf]

Structure-function analysis of PorX$_{Fj}$, the PorX homolog from *Flavobacterium johnsioniae*, suggests a role of the CheY-like domain in type IX secretion motor activity

Mariotte Zamitt[1], Julia Bartoli[1], Christine Kellenberger[2], Pauline Melani[1], Alain Roussel[1], Eric Cascales[1] and Philippe Leone[1,*]

[1] Laboratoire d'Ingénierie des Systèmes Macromoléculaires (LISM, UMR7255), Institut de Microbiologie de la Méditerranée, Aix Marseille Univ, Centre National de la Recherche Scientifique, Marseille, France
[2] Laboratoire de Chimie Bactérienne (LCB, UMR7283), Institut de Microbiologie de la Méditerranée, Aix Marseille Univ, Centre National de la Recherche Scientifique, Marseille, France

[*] Correspondence : pleone@imm.cnrs.fr

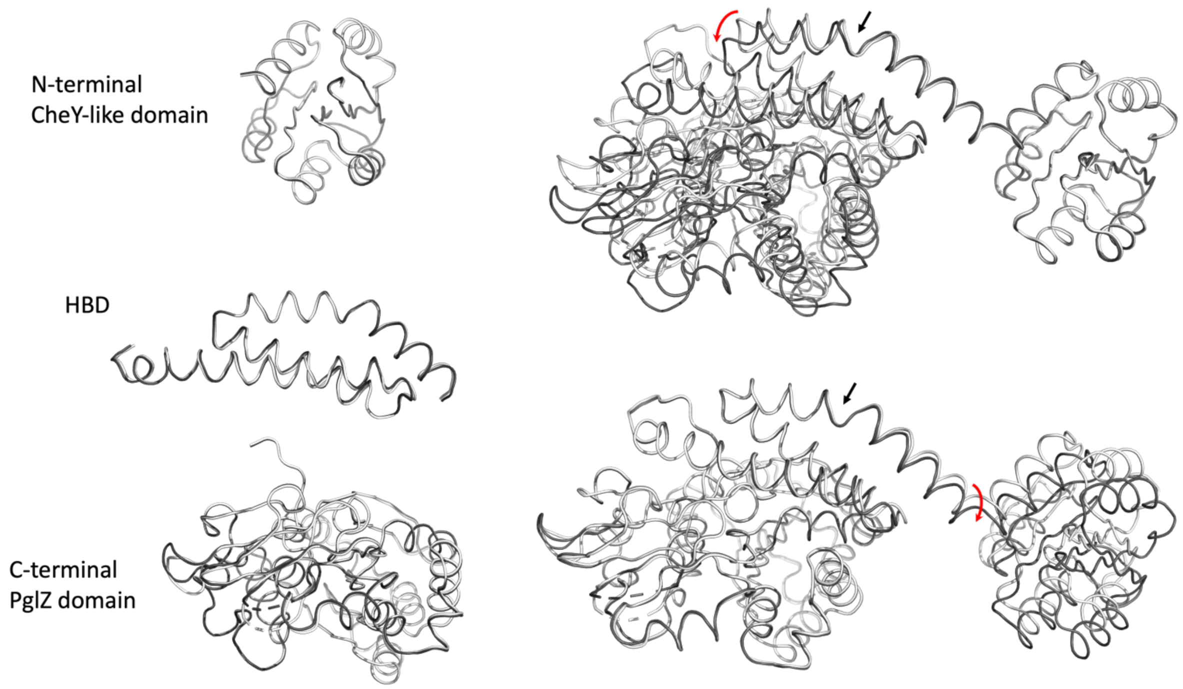

Supplementary Fig. 1 Comparison of the two PorX$_{Fj}$ molecules present in the asymmetric unit.
Left: the three PorX$_{Fj}$ domains of each molecule are independently superimposed. Right: the two whole PorX$_{Fj}$ molecules are superimposed through their N-terminal (top) and C-terminal (bottom) domains.
Molecules A and B are colored in light and dark grey, respectively; the red arrows highlight the shift of the HBD's first helix, and the black arrow shows the kink present in this helix.

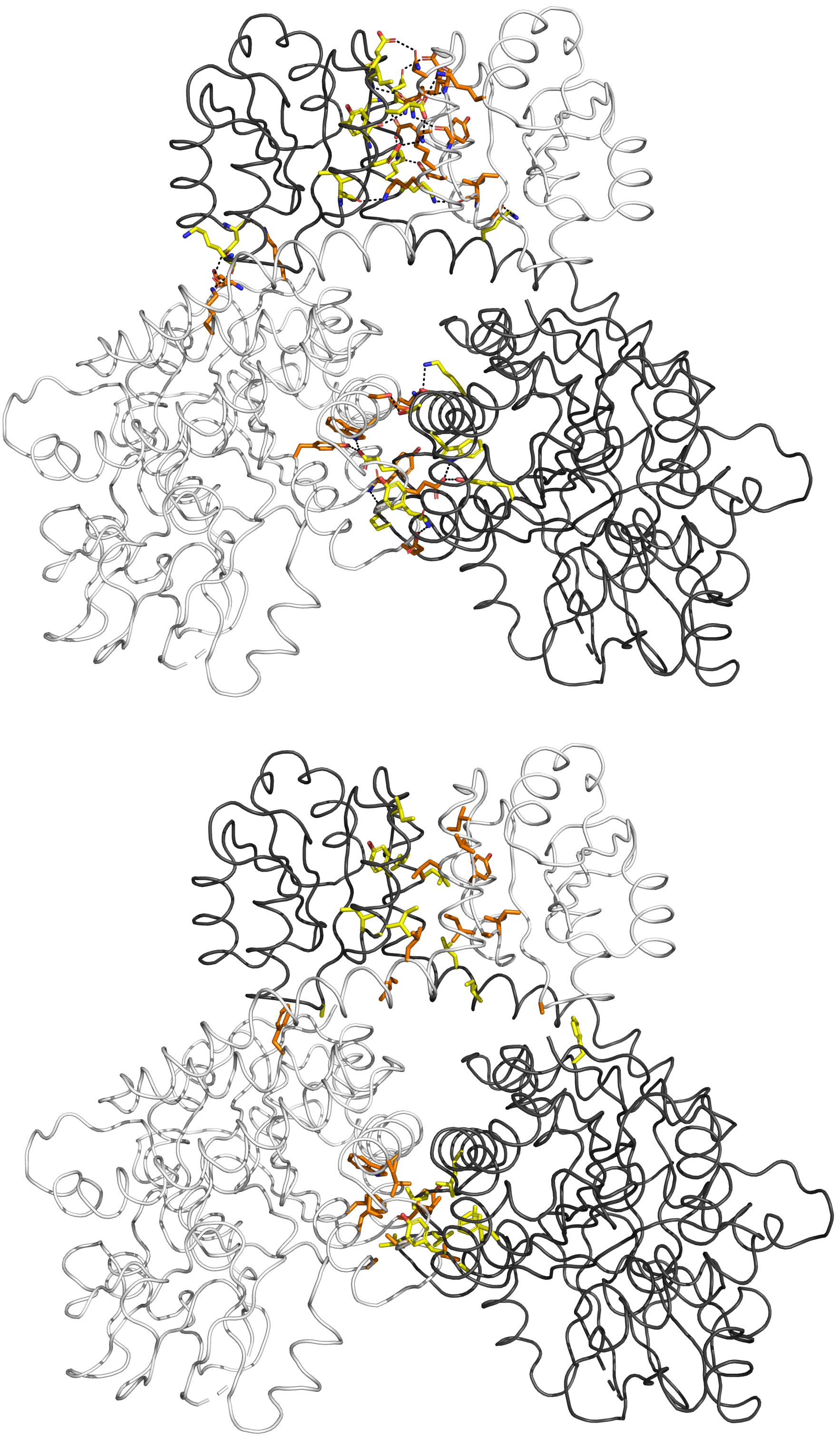

Supplementary Fig. 2 : Interactions network at the interface between the two $PorX_{Fj}$ molecules present in the asymmetric unit.

Top: electrostatic interactions (within a distance of 3.2 Å). Bottom: hydrophobic contacts (within a distance of 4.5 Å between hydrophobic residues from each molecule)

Molecules A and B are colored in light and dark grey, respectively, and their corresponding residues are colored in orange and yellow, respectively; hydrogen bonds are displayed as black dashed lines. Residues are listed in Table S1.

Supplementary Table 1: Residues in contact at the interface between the two $PorX_{Fj}$ molecules present in the asymmetric unit.

Residues are displayed in Fig. S2.

molecule A	molecule B
Electrostatic interactions	
Hydrogen bonds	
Glu85 OE1	Asn109 OD1
Ser96 OG	Lys128 NZ
Ile98 O	Lys117 NZ
Tyr101 O	Gln110 NE2
Asn109 OD1	Glu85 OE1
Gln110 NE2	Tyr101 O
OE1	Ile103 N
Ile103 N	Gln110 OE1
Lys117 NZ	Ile98 O
Lys158 NZ	Lys72 O
Lys256 NZ	Asn47 O
Glu258 O	Asn47 ND2
Tyr333 N	Glu370 OE2
Tyr356 OH	Glu374 OE2
Glu370 OE2	Tyr333 N
Glu374 OE2	Tyr356 OH
	Trp389 NE1
Ser384 OG	Ser388 OG
Ser388 OG	Ser384 OG
Trp389 NE1	Glu374 OE2
Asn392 OD1	Lys380 NZ
Salt bridges	
Lys26 NZ	Glu87 OE1
Glu87 OE1	Lys26 NZ
	Lys116 NZ
Glu91 OE2	Lys116 NZ
Lys116 NZ	Glu87 OE2
	Glu91 OE2
Hydrophobic residues	
Ala75	
Ile94	
Ile98	
Ala99	
Tyr101	
Ile103	
Leu112	
Leu113	
Leu124	
Ile125	
Phe162	
Tyr333	
Phe358	

Leu362
Ala365
Val371
Val372
Leu375
Ala381
Leu385
Trp389

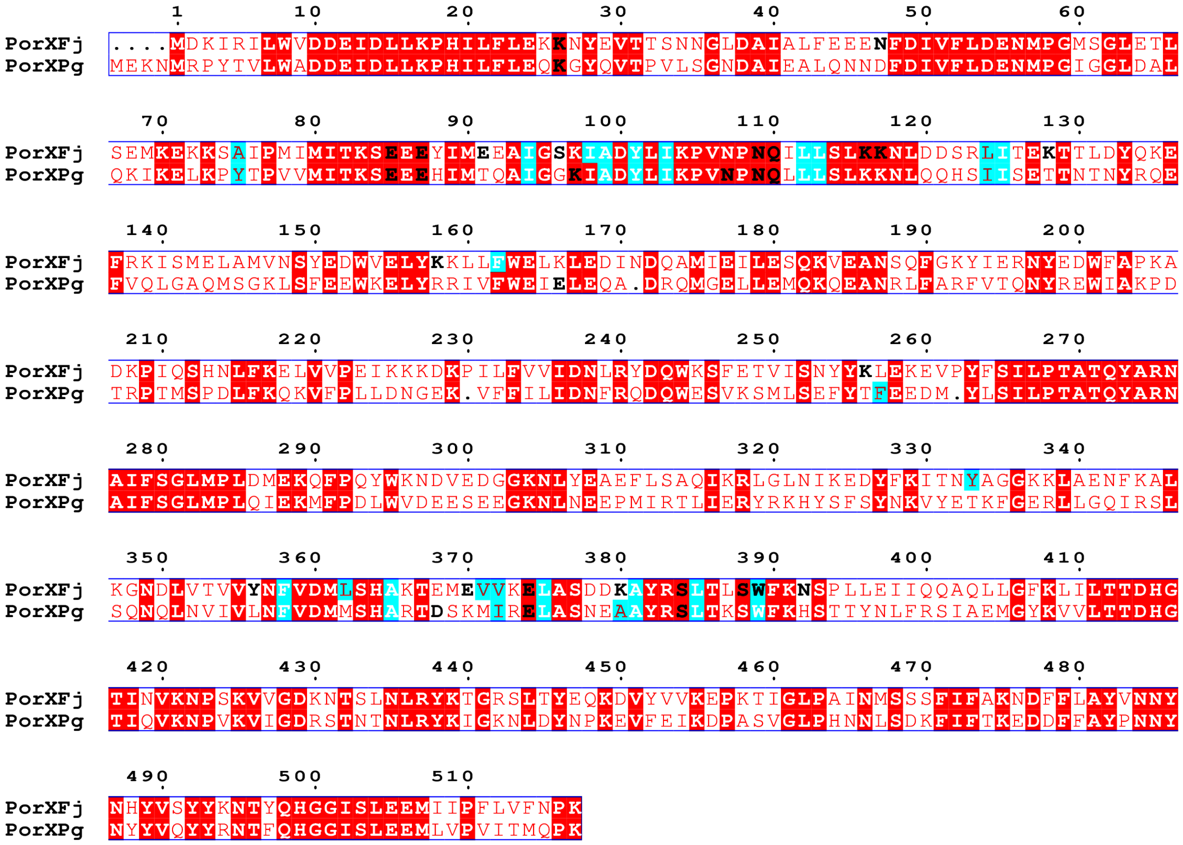

Supplementary Fig. 3 : Sequence alignment of PorX$_{Fj}$ and PorX$_{Pg}$.

PorX$_{FJ}$ and Por$_{Pg}$ residues whose side chain is involved in electrostatic interactions or hydrophobic contacts at the dimer interface are labelled in black and boxed in cyan, respectively. Reference amino-acid numbering is according to the PorX$_{FJ}$ sequence.
